# Association of high-risk CT coronary artery plaque features with major adverse cardiovascular events: a prespecified secondary analysis of the DISCHARGE trial

**DOI:** 10.1007/s00330-025-12146-3

**Published:** 2026-02-13

**Authors:** Bálint Szilveszter, Federico Biavati, Charlotte Sørum, Theodora Benedek, Patrick Donnelly, José F. Rodriguez-Palomares, Andrejs Erglis, Cyril Štěchovský, Gintarė Šakalytė, Nada Čemerlić Ađić, Matthias Gutberlet, Jonathan D. Dodd, Ignacio Diez, Gershan Davis, Elke Zimmermann, Cezary Kepka, Radosav Vidakovic, Marco Francone, Małgorzata Ilnicka-Suckiel, Fabian Plank, Juhani Knuuti, Rita Faria, Stephen Schröder, Colin Berry, Luca Saba, Balazs Ruzsics, Nina Rieckmann, Christine Kubiak, Kristian Schultz Hansen, Jacqueline Müller-Nordhorn, Pal Maurovich-Horvat, Helena Domínguez Vall-Lamora, Imre Benedek, Clare Orr, Filipa Valente, Ligita Zvaigzne, Vojtěch Suchánek, Antanas Jankauskas, Filip Ađić, Michael Woinke, Niall Mulvihill, Iñigo Lecumberri, Erica Thwaite, Mariusz Kruk, Aleksandar N. Neskovic, Massimo Mancone, Donata Kuśmierz, Gudrun Maria Feuchtner, Mikko Pietilä, Vasco Gama Ribeiro, Tanja Drosch, Christian Delles, Alberto Boi, Michael Fisher, Béla Merkely, Thomas Fritz Hansen, Rosca Aurelian, Stephanie Kelly, Bruno García del Blanco, Ainhoa Rubio, Melinda Boussoussou, Tem Jørgensen, Ioana Rodean, Susan Regan, Hug Cuéllar Calabria, Borbála Vattay, Roxana Hodas, Adriane Elisabeth Napp, Robert Haase, Sarah Feger, Mahmoud M. A. Mohamed, Lina M. Serna-Higuita, Konrad Neumann, Henryk Dreger, Matthias Rief, Viktoria Wieske, Michelle C. Williams, Melanie Estrella, Maria Bosserdt, Peter Martus, Klaus F. Kofoed, Marc Dewey

**Affiliations:** 1 Heart and Vascular Center, Semmelweis University, Budapest, Hungary; 2 Department of Radiology, Charité–Universitätsmedizin Berlin, Freie Universität Berlin and Humboldt-Universität zu Berlin, Berlin, Germany; 3 Department of Cardiology, Copenhagen University Hospital, Bispebjerg and Frederiksberg, Frederiksberg, Denmark; 4 Department of Internal Medicine, Clinic of Cardiology, George Emil Palade University of Medicine, Pharmacy, Science and Technology, Targu Mures, Romania; 5 County Clinical Emergency Hospital Targu Mures, Targu Mures, Romania; 6 Department of Cardiology, Southeastern Health and Social Care Trust, Belfast, United Kingdom; 7 Department of Cardiology, Hospital Universitario Vall d’Hebron, Institut de Recerca (VHIR), Universitat Autònoma de Barcelona, Barcelona, Spain; 8 Centro de Investigacion Biomedica en Red, Madrid, Spain; 9 Department of Cardiology, Paul Stradins Clinical University Hospital, Riga, Latvia; 10 University of Latvia, Riga, Latvia; 11 Department of Cardiology, Motol University Hospital, Prague, Czech Republic; 12 Department of Cardiology, Medical Academy, Lithuanian University of Health Sciences, Kaunas, Lithuania; 13 Department of Cardiology, Hospital of Lithuanian University of Health Sciences, Kaunas, Lithuania; 14 Faculty of Medicine, University of Novi Sad, Novi Sad, Serbia; 15 Department of Cardiology, Institute for Cardiovascular Diseases of Vojvodina, Novi Sad, Serbia; 16 Department of Radiology, University of Leipzig Heart Centre, Leipzig, Germany; 17 Department of Radiology, St. Vincent’s University Hospital, Dublin, Ireland; 18 School of Medicine, University College Dublin, Dublin, Ireland; 19 Department of Cardiology, Basurto Hospital, Bilbao, Spain; 20 Department of Cardiology, Aintree University Hospital, Liverpool, United Kingdom; 21 Edge Hill University, Ormskirk, United Kingdom; 22 National Institute of Cardiology, Warsaw, Poland; 23 Department of Cardiology, Internal Medicine Clinic, Clinical Hospital Center Zemun, Belgrade, Serbia; 24 Faculty of Medicine, University of Belgrade, Belgrade, Serbia; 25 Department of Radiological, Oncological and Pathological Sciences, Sapienza University of Rome, Rome, Italy; 26 Department of Biomedical Sciences, Humanitas University and IRCCS Humanitas Research Hospital, Milan, Italy; 27 Department of Cardiology, Provincial Specialist Hospital in Wroclaw, Wroclaw, Poland; 28 Department of Internal Medicine III, Department of Cardiology, Innsbruck Medical University, Innsbruck, Austria; 29 Turku PET Centre, Turku University Hospital and University of Turku, Turku, Finland; 30 Department of Cardiology, Centro Hospitalar de Vila Nova de Gaia-Espinho, Vila Nova de Gaia, Portugal; 31 Department of Cardiology, Alb Fils Kliniken, Göppingen, Germany; 32 School of Cardiovascular and Metabolic Health, University of Glasgow, Glasgow, United Kingdom; 33 Golden Jubilee National Hospital, Clydebank, United Kingdom; 34 Department of Radiology, University of Cagliari, Cagliari, Italy; 35 Department of Cardiology, Liverpool University Hospital NHS FT, Liverpool, United Kingdom; 36 Institute for Cardiovascular Medicine and Science, Liverpool Heart and Chest Hospital, Liverpool, United Kingdom; 37 Institute of Public Health, Charité–Universitätsmedizin Berlin, Freie Universität Berlin and Humboldt-Universität zu Berlin, Berlin, Germany; 38 ECRIN-ERIC (European Clinical Research Infrastructure Network-European Research Infrastructure Consortium), Paris, France; 39 Department of Public Health, Section for Health Services Research, University of Copenhagen, Copenhagen, Denmark; 40 Bavarian Cancer Registry, Bavarian Health and Food Safety Authority, Munich, Germany; 41 Department of Radiology, Medical Imaging Center, Semmelweis University, Budapest, Hungary; 42 Department of Biomedicine, University of Copenhagen, Copenhagen, Denmark; 43 Center of Advanced Research in Multimodality Cardiac Imaging, CardioMed Medical Center, Targu Mures, Romania; 44 Department of Radiology, Hospital Universitario Vall d’Hebron, Institut de Recerca, Universitat Autònoma de Barcelona, Barcelona, Spain; 45 Department of Radiology, Paul Stradins Clinical University Hospital, Riga, Latvia; 46 Department of Imaging Methods, Motol University Hospital, Prague, Czech Republic; 47 Institute of Cardiology, Lithuanian University of Health Sciences, Department of Radiology, Kaunas Clinics, Kaunas, Lithuania; 48 Department of Cardiology, University of Leipzig Heart Centre, Leipzig, Germany; 49 Department of Cardiology, St. Vincent’s University Hospital, Dublin, Ireland; 50 Department of Radiology, Basurto Hospital, Bilbao, Spain; 51 Department of Radiology, Aintree University Hospital, Liverpool, United Kingdom; 52 Department of Clinical Internal, Anesthesiologic and Cardiovascular Sciences, Sapienza University of Rome, Rome, Italy; 53 Department of Radiology, Provincial Specialist Hospital in Wroclaw, Wroclaw, Poland; 54 Department of Radiology, Innsbruck Medical University, Innsbruck, Austria; 55 Heart Center, Turku University Hospital and University of Turku, Turku, Finland; 56 Administrative Centre, Health Care District of Southwestern Finland, Turku, Finland; 57 Department of Cardiology, Azienda Ospedaliera Brotzu, Cagliari, Italy; 58 Liverpool Centre for Cardiovascular Science, University of Liverpool, Liverpool, United Kingdom; 59 Department of Cardiology, Copenhagen University Hospital - Herlev and Gentofte, Copenhagen, Denmark; 60 Department of Cardiology, George Emil Palade University of Medicine, Pharmacy, Science and Technology, Tirgu Mures, Romania; 61 Department of Cardiology, Amager-Hvidovre Hospital, University of Copenhagen, Copenhagen, Denmark; 62 Department of Clinical Epidemiology and Applied Biostatistics, Universitätsklinikum Tübingen, Tübingen, Germany; 63 Institute of Biometry and Clinical Epidemiology, Charité–Universitätsmedizin Berlin, Freie Universität Berlin and Humboldt-Universität zu Berlin, Berlin, Germany; 64 Department of Cardiology and Angiology, Charité–Universitätsmedizin Berlin, Freie Universität Berlin and Humboldt-Universität zu Berlin, Berlin, Germany; 65 DZHK (German Centre for Cardiovascular Research), partner site Berlin, Berlin, Germany; 66 British Heart Foundation Centre for Cardiovascular Science, University of Edinburgh, Edinburgh, Scotland; 67 Department of Cardiology, Copenhagen University Hospital - Rigshospitalet & Department of Clinical Medicine, Faculty of Health and Medical Sciences, University of Copenhagen, Copenhagen, Denmark; 68 Department of Radiology, Copenhagen University Hospital - Rigshospitalet & Department of Clinical Medicine, Faculty of Health and Medical Sciences, University of Copenhagen, Copenhagen, Denmark; 69 Deutsches Herzzentrum der Charité (DHZC), Department of Cardiology, Angiology and Intensive Care Medicine, Campus Charité Mitte, Berlin, Germany; 70 Berlin University Alliance, Berlin, Germany

**Keywords:** Angina pectoris, Computed tomography, Coronary artery disease, Prognosis

## Abstract

**Objectives:**

The prognostic role of high-risk plaque (HRP) features, including high coronary calcium scores detected by CT, beyond traditional cardiovascular risk factors and obstructive coronary artery disease (CAD), remains uncertain. This study evaluated the prognostic value of a combined HRP definition in stable chest pain patients with low-to-intermediate pretest probability of CAD.

**Materials and methods:**

This prespecified analysis included participants randomized to the CT arm of the pragmatic, prospective 26-center European DISCHARGE trial (NCT02400229). The primary endpoint was major adverse cardiovascular events (MACE: cardiovascular death, nonfatal myocardial infarction, or stroke); the secondary endpoint was expanded MACE (transient ischemic attack and major procedure-related complications). Our combined HRP definition was any coronary plaque with positive remodeling, napkin-ring sign, low attenuation, or total calcium score ≥ 400 Agatston units.

**Results:**

Among 1745 participants (age: 60 ± 10 years, 990 female), 35 MACE and 47 expanded MACE occurred at a median follow-up of 3.5 years (IQR: 2.9–4.2). After risk factor adjustment, the combined HRP definition was associated with a higher risk of MACE (HR: 3.81; 95% CI: 1.01–14.6; *p* = 0.050) and remained significantly associated with expanded MACE (HR: 0.21; 95% CI: 1.07–9.66; *p* = 0.038). Patients with both HRP and obstructive CAD conferred the highest MACE (HR: 8.78; 95% CI: 2.90–26.6; *p* < 0.001) and expanded MACE risk (HR: 7.31; 95% CI: 3.06–17.48; *p* < 0.001) compared with patients without HRP or obstructive CAD. HRP alone showed a prognostic impact comparable to obstructive CAD alone.

**Conclusion:**

HRP features on coronary CT provide incremental predictive value for MACE, comparable to obstructive CAD, after adjusting for traditional cardiovascular risk factors in this large pan-European cohort.

**Trial registration:**

ClinicalTrials.gov NCT02400229.

**Key Points:**

***Question*** *Do high-risk plaque (HRP) features on coronary CT have incremental prognostic value after adjustment for cardiovascular risk factors in stable chest pain?*

***Findings*** *A combined definition of HRP features on coronary CT was independently associated with major adverse cardiovascular events, with similar prognostic value to obstructive coronary artery disease after risk adjustment.*

***Clinical relevance*** *HRP features, including a coronary artery calcium score ≥ 400 Agatston units or traditional high-risk plaque characteristics on coronary CT, provide incremental prognostic value in predicting MACE in stable chest pain patients, offering a potential tool for improved risk stratification and management.*

**Graphical Abstract:**

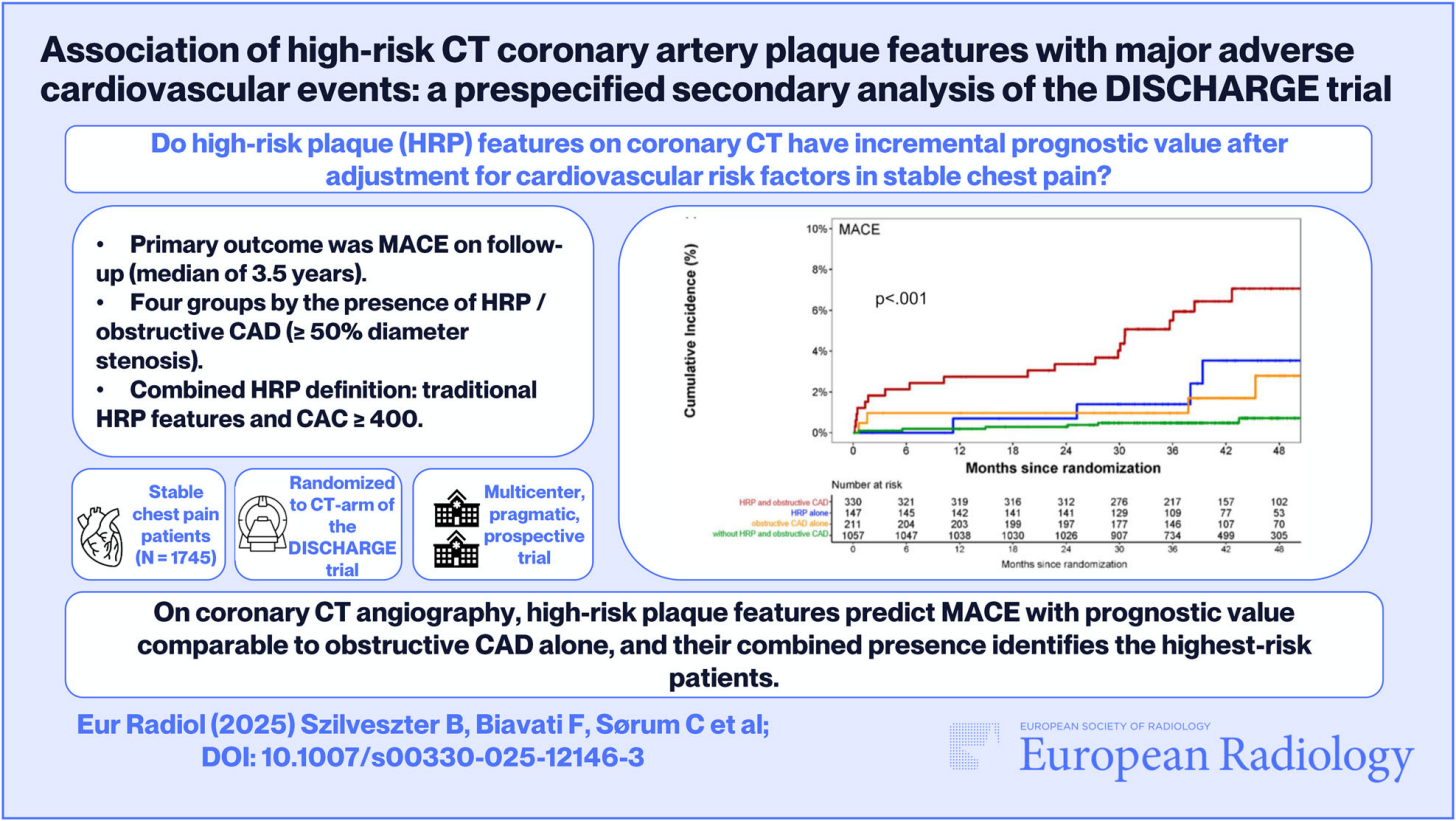

## Introduction

Coronary computed tomography (CT) provides a comprehensive assessment of coronary artery disease (CAD), including its extent, location, and severity, as well as the presence of high-risk plaque (HRP) characteristics [1]. High coronary calcium scores—reflecting total coronary plaque burden—remain a limitation in identifying HRP on cardiac CT [2, 3]. Recent trials identified CT angiography (CTA) as a safe, noninvasive gatekeeper to invasive coronary angiography (ICA) [4–6]. While traditional assessment of stable angina patients focused on the functional evaluation of CAD and the detection of luminal stenosis, recent guidelines, such as the 2021 AHA/ACC guideline on chest pain [7], also recommend CT imaging in this population. Major adverse cardiovascular events (MACE) can occur in patients with nonobstructive CAD; therefore, adverse plaque characteristics may help refine risk assessment beyond luminal stenosis [8]. In the Scottish Computed Tomography of the Heart (SCOT-HEART) trial, CT improved diagnostic certainty and altered subsequent patient management, ultimately reducing the mortality risk of patients referred to cardiology for as

The multicenter, randomized DISCHARGE (Diagnostic Imaging Strategies for Patients with Stable Chest Pain and Intermediate Risk of Coronary Artery Disease) trial compared CT with ICA as initial diagnostic imaging strategies for guiding the management of participants with stable chest pain and low-to-intermediate pretest probability (10–60%) of obstructive CAD referred for ICA [9]. The frequency of major procedure-related complications in the DISCHARGE trial was lower in the initial-CTA group than in the initial-ICA group, while MACE rates were similar at a median follow-up of 3.5 years. Regarding gender differences, an initial CT scan was associated with fewer major procedure-related complications in women and a lower rate of the expanded MACE composite in men [10]. In prior studies, both the extent of CAD based on coronary artery calcium (CAC) scores and traditional HRP characteristics as detected by CT have been associated with MACE, and the identification of these features has the potential to optimize patient management [11–13]. It remains unclear whether these are independent prognostic factors with an incremental value over traditional cardiovascular (CV) risk factors or obstructive CAD.

In this substudy of the DISCHARGE trial, we aimed to elucidate whether a novel HRP definition combining traditional HRP features with high CAC scores was associated with MACE and an expanded MACE composite including transient ischemic attack (TIA) and major periprocedural complications in the presence and absence of obstructive CAD and conventional CV risk factors in patients with stable chest pain initially referred for ICA but assigned to the CT arm of the trial.

## Materials and methods

### Study population

**Fig. 1:**
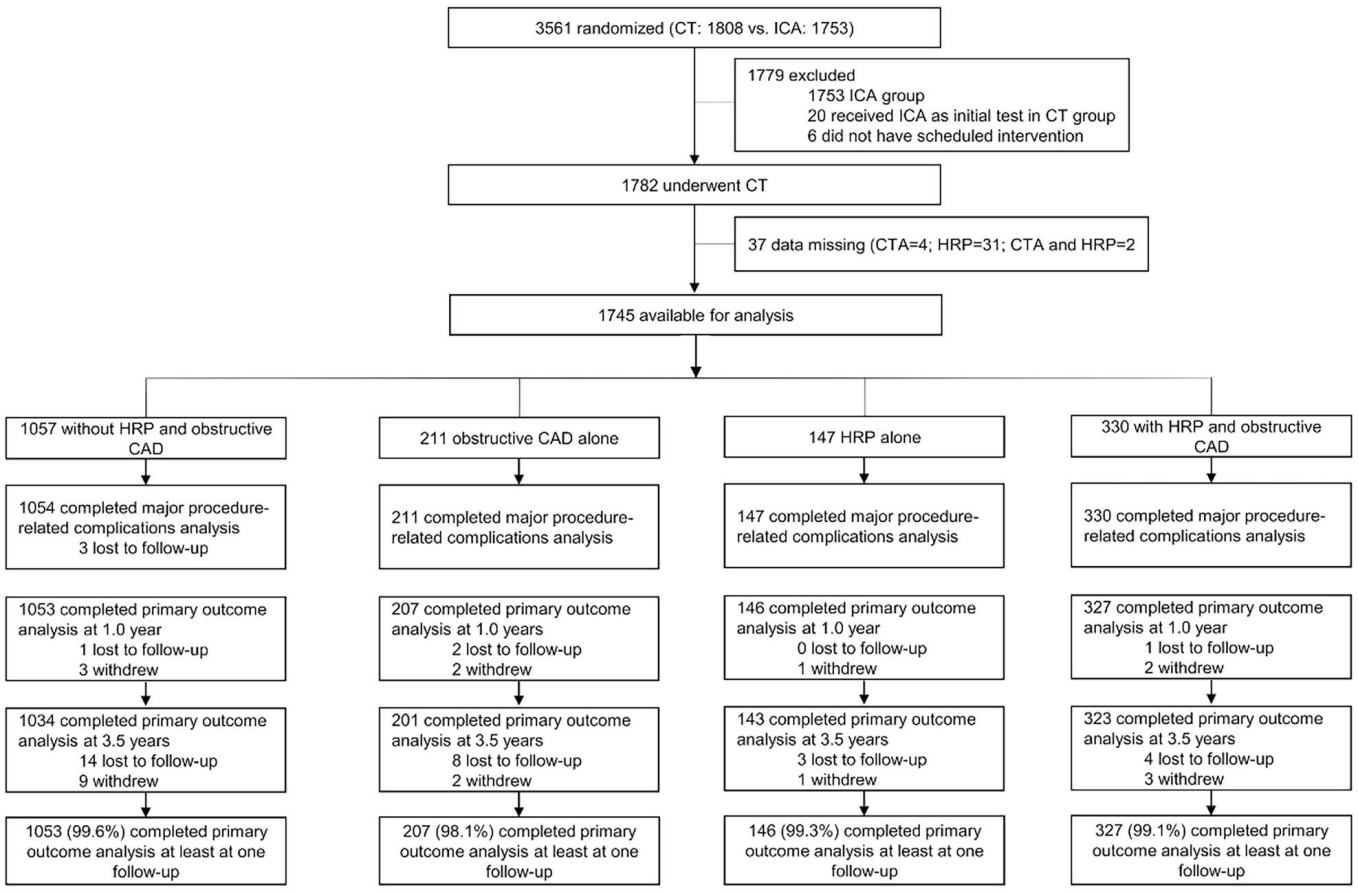
Consort diagram. Flowchart of patient inclusion in the substudy of HRP features. We analyzed 1745 participants assigned to one of four groups based on the presence of the combined HRP definition and obstructive CAD in the CT arm of the trial. CAD, coronary artery disease; CT, computed tomography; HRP, high-risk plaque; ICA, invasive coronary angiography

The study was conducted in accordance with local and federal regulations and the Declaration of Helsinki. Written informed consent was obtained from all participants. In this prespecified subgroup analysis of the prospective, pragmatic, multicenter, pan-European randomized DISCHARGE trial (NCT02400229, #100, Statistical Analysis Plan in Table 19), we analyzed 1782 of 3561 participants with suspected CAD, low-to-intermediate risk, and stable chest pain who underwent CT in an intention-to-treat population (Fig. 1). Details on the design, inclusion and exclusion criteria, data management, and the main results of the trial are described in recent publications along with the list of participating clinical centers [9, 14]. Participant demographics, co-morbidities, risk factors, and data on prior testing were recorded during enrollment and are summarized in Tables 1 and 2. Further details on randomization, patient characteristics are summarized in Supplementary Table 1.

**Table 1:** Baseline characteristics stratified by HRP features and obstructive CAD in participants who underwent CT (*n* = 1745)

	Without HRP and obstructive CAD	Obstructive CAD alone	Combined HRP criteria only	With HRP and obstructive CAD	*p*-value
	(*N* = 1057)	(*N* = 211)	(*N* = 147)	(*N* = 330)	
Age (years)	58.5 (10.3)	60.0 (9.98)	62.2 (9.63)	64.8 (8.64)	< 0.001^Anova^
Women	665 (62.9%)	120 (56.9%)	73 (49.7%)	132 (40.0%)	< 0.001^Chi2^
Men	392 (37.1%)	91 (43.1%)	74 (50.3%)	198 (60.0%)	
In- and outpatients at the time of trial enrollment					
Inpatients	193 (18.3%)	59 (28.0%)	32 (21.8%)	72 (21.8%)	< 0.01^Chi2^
Outpatients	836 (79.1%)	143 (67.8%)	109 (74.1%)	246 (74.5%)	
Missing	28 (2.6%)	9 (4.3%)	6 (4.1%)	12 (3.6%)	
Pretest probability‡	35.2 (10.6)	38.0 (11.0)	39.1 (10.3)	43.0 (9.84)	< 0.001^Anova^
Type of chest pain					
Typical angina	150 (14.2%)	34 (16.1%)	11 (7.5%)	34 (10.3%)	0.33^LL^
Atypical angina	477 (45.1%)	100 (47.4%)	83 (56.5%)	151 (45.8%)	
Nonanginal chest pain	397 (37.6%)	74 (35.1%)	50 (34.0%)	133 (40.3%)	
Other chest pain	33 (3.1%)	3 (1.4%)	3 (2.0%)	12 (3.6%)	
ICA referral categories§					0.66^LL^
Clinical constellation suggesting high event risk, particularly with inadequate response to medical treatment	531 (50.2%)	85 (40.3%)	75 (51.0%)	142 (43.0%)	
Severe angina, particularly with inadequate response to medical treatment	192 (18.2%)	39 (18.5%)	35 (23.8%)	74 (22.4%)	
Intermediate pretest probability of CAD or LVEF < 50% without typical angina after functional testing showing ischemia	144 (13.6%)	46 (21.8%)	18 (12.2%)	61 (18.5%)	
Low or intermediate event risk with inadequate response to medical treatment	109 (10.3%)	27 (12.8%)	11 (7.5%)	40 (12.1%)	
Intermediate pretest probability or LVEF < 50% without typical angina after nondiagnostic functional testing	32 (3.0%)	7 (3.3%)	5 (3.4%)	8 (2.4%)	
Other‖‖	44 (4.2%)	7 (3.3%)	3 (2.0%)	5 (1.5%)	
Missing	5 (0.5%)	0 (0%)	0 (0%)	0 (0%)	
Cardiovascular risk factors					
Arterial hypertension	595 (56.3%)	132 (62.6%)	100 (68.0%)	243 (73.6%)	< 0.001^Chi2^
Diabetes mellitus	116 (11.0%)	35 (16.6%)	24 (16.3%)	73 (22.1%)	< 0.001^Chi2^
Hyperlipidemia	450 (42.6%)	119 (56.4%)	74 (50.3%)	195 (59.1%)	< 0.001^Chi2^
Missing	6 (0.6%)	0 (0%)	0 (0%)	0 (0%)	
Peripheral artery disease	8 (0.8%)	1 (0.5%)	3 (2.0%)	9 (2.7%)	0.02^Fish^
Valve disease	50 (4.7%)	9 (4.3%)	9 (6.1%)	25 (7.6%)	0.20^Chi2^
Stroke	24 (2.3%)	4 (1.9%)	3 (2.0%)	13 (3.9%)	0.38^Fish^
Transient ischemic attack (TIA)	17 (1.6%)	3 (1.4%)	0 (0%)	9 (2.7%)	0.18^Fish^
Prolonged ischemic neurological deficit	2 (0.2%)	0 (0%)	0 (0%)	0 (0%)	
Carotid artery disease	7 (0.7%)	0 (0%)	10 (6.8%)	17 (5.2%)	< 0.001^Fish^
Family history of premature CAD (women)	211/660 (32.0%)	50/120 (41.7%)	15/73 (20.5%)	33/131 (25.2%)	
Family history of premature CAD (men)	98/391 (25.1%)	32/91 (35.2%)	15/74 (20.3%)	39/198 (19.7%)	
Missing	6 (0.6%)	0 (0%)	0 (0%)	1 (0.3%)	
Pulmonary risk factors					
Asthma	80 (7.6%)	9 (4.3%)	10 (6.8%)	20 (6.1%)	0.32^Chi2^
Chronic obstructive pulmonary disease	42 (4.0%)	6 (2.8%)	12 (8.2%)	10 (3.0%)	0.04^Chi2^
Missing	6 (0.6%)	0 (0%)	0 (0%)	1 (0.3%)	
Cigarette smokers					0.27^Chi2^
Current smokers	198 (18.7%)	37 (17.5%)	34 (23.1%)	59 (17.9%)	
Former smokers	302 (28.6%)	64 (30.3%)	42 (28.6%)	116 (35.2%)	
Never smoked	523 (49.5%)	105 (49.8%)	67 (45.6%)	144 (43.6%)	
Missing	34 (3.2%)	5 (2.4%)	4 (2.7%)	11 (3.3%)	
BMI (kg/m^2^)	28.9 (5.20)	29.3 (5.09)	28.5 (4.83)	28.7 (4.68)	0.51^Anova^
Missing	34 (3.2%)	11 (5.2%)	5 (3.4%)	9 (2.7%)	
Cardiovascular medications					
Statin	409 (38.7%)	112 (53.1%)	69 (46.9%)	183 (55.5%)	< 0.001^Chi2^
Antiplatelet agent	438 (41.4%)	124 (58.8%)	65 (44.2%)	194 (58.8%)	< 0.001^Chi2^
Beta-blocker	402 (38.0%)	107 (50.7%)	69 (46.9%)	151 (45.8%)	< 0.01^Chi2^
Nitrates	96 (9.1%)	37 (17.5%)	6 (4.1%)	49 (14.8%)	< 0.001^Chi2^
Calcium antagonist	189 (17.9%)	41 (19.4%)	38 (25.9%)	85 (25.8%)	< 0.01^Chi2^
Angiotensin-converting enzyme inhibitor or angiotensin-receptor blocker	453 (42.9%)	108 (51.2%)	86 (58.5%)	199 (60.3%)	< 0.001^Chi2^
Missing	2 (0.6%)	0 (0%)	1 (0.5%)	10 (0.9%)	
Functional test performed before the assigned intervention	350 (33.1%)	81 (38.4%)	54 (36.7%)	100 (30.3%)	0.21^Chi2^
Positive results	144 (13.6%)	46 (21.8%)	18 (12.2%)	61 (18.5%)	< 0.01^Chi2^
Negative results	174 (16.5%)	28 (13.3%)	31 (21.1%)	31 (9.4%)	
Nondiagnostic results	32 (3.0%)	7 (3.3%)	5 (3.4%)	8 (2.4%)	
Physical component score (SF12v2)	44.3 (9.17)	43.8 (8.79)	43.7 (9.30)	43.6 (8.79)	0.61 ^Anova^

Values are means (SDs) unless otherwise stated. Data on cardiovascular medications reflect treatments documented at screening

*ANOVA* analysis of variance, *BMI* body mass index, *CAD* coronary artery disease, *Chi2* Chi-square test, *CT* computed tomography, *Fish* Fisher test, *ICA* invasive coronary angiography, *LL* linear-by-linear association test, *LVEF* left ventricular ejection fraction, *SD* standard deviation, *TIA* transient ischemic attack

‡ Calculated pretest probability of CAD using an automated calculation, integrated into a web-based system of the electronic case report forms, which applied an updated model of the Diamond and Forrester method using participants‘ age, gender, and type of stable chest pain

§ ICA referral categories were defined according to the European guidelines for management of stable CAD

‖‖ Other ICA referral categories included a lack of ability to undergo stress imaging (11 in participants without HRP and obstructive CAD, 0 in participants with obstructive CAD alone, 0 in participants with HRP alone and 1 in participants with HRP and obstructive CAD), an LVEF level of less than 50% and typical angina (11 in participants without HRP and obstructive CAD, 3 in participants with obstructive CAD alone, 0 in participants with HRP alone and 0 participants with HRP and obstructive CAD), the presence of mild symptoms with medical treatment with noninvasive risk stratification indicating a high event risk and consideration of revascularization for improvement of prognosis (17 in participants without HRP and obstructive CAD, 3 in participants with obstructive CAD alone, 5 in participants with HRP alone and 2 participants with HRP and obstructive CAD), inconclusive diagnosis on noninvasive testing or conflicting results from different noninvasive methods (4 in participants without HRP and obstructive CAD, 1 in participants with obstructive CAD alone, 0 in participants with HRP alone and 0 in participants with HRP and obstructive CAD), and employment in a special profession, such as airplane pilot, due to regulatory issues (1 in participants without HRP and obstructive CAD, 0 in participants with obstructive CAD alone, 0 in participants with HRP alone and 0 in participants with HRP and obstructive CAD)

**Table 2:** Comparison of diagnostic and treatment strategies and coronary findings across subgroups based on CT findings

	Without HRP and obstructive CAD	Obstructive CAD alone	Combined HRP criteria only	With HRP and obstructive CAD	*p*-value
	(*N* = 1057)	(*N* = 211)	(*N* = 147)	(*N* = 330)	
Time from enrollment to initial test (IQR) in days*	3.00 (0–14)	2.00 (0–15)	7.00 (0–16)	3.00 (0–12)	0.35^KW^
Diagnostic findings on CT					
Obstructive CAD: ≥ 50% stenosis	0 (0%)	139 (65.9%)	0 (0%)	304 (92.1%)	
1 vessel	0 (0%)	57 (27.0%)	0 (0%)	89 (27.0%)	
2 vessels	0 (0%)	22 (10.4%)	0 (0%)	33 (10.0%)	
High-risk anatomy†	0 (0%)	60 (28.4%)	0 (0%)	182 (55.2%)	
Nonobstructive CAD: 1–49% stenosis	500 (47.3%)	0 (0%)	147 (100%)	0 (0%)	
No sign of CAD	557 (52.7%)	0 (0%)	0 (0%)	0 (0%)	
Nondiagnostic results‡	0 (0%)	72 (34.1%)	0 (0%)	26 (7.9%)	
Positive remodeling	0 (0%)	0 (0%)	53 (36.1%)	39 (11.8%)	< 0.001 ^LL^
Low CT attenuation	0 (0%)	0 (0%)	74 (50.3%)	136 (41.2%)	< 0.001 ^LL^
Napkin-ring sign	0 (0%)	0 (0%)	43 (29.3%)	110 (33.3%)	< 0.001 ^LL^
CAC score class					
CAC score: 0	652 (61.7%)	63 (29.9%)	25 (17.0%)	13 (3.9%)	< 0.001^LL^
CAC score: 1–399	405 (38.3%)	148 (70.1%)	81 (55.1%)	108 (32.7%)	
CAC score: ≥ 400	0 (0%)	0 (0%)	41 (27.9%)	209 (63.3%)	
Mean CAC score	25.2 (58.4)	95.4 (109)	289 (453)	789 (844)	< 0.001^Anova^
ICA performed during initial management	6 (0.6%)	109 (51.7%)	3 (2.0%)	251 (76.1%)	< 0.001^LL^
PCI during initial management	0 (0%)	47 (22.3%)	0 (0%)	138 (41.8%)	< 0.001^Fish^
CABG during initial management	0 (0%)	4 (1.9%)	0 (0%)	33 (10.0%)	< 0.001^Fish^

Values are means (SDs) unless otherwise stated

*ANOVA* analysis of variance, *CABG* coronary artery bypass graft, *CAC* coronary artery calcium, *CAD* coronary artery disease, *Chi2* Chi-square test, *Fish* Fisher test, *KW* Kruskal–Wallis test, *LL* linear-by-linear association test, *IQR* interquartile range, *CT* computed tomography, *ICA* invasive coronary angiography, *PCI* percutaneous coronary intervention

* Times to initial test results are cumulative incidence estimates

† High-risk anatomy CAD was defined by the initial test as any 3-vessel CAD or left main coronary artery stenosis or proximal left anterior descending coronary artery stenosis or any combination of these

‡ Nondiagnostic test was defined as a relevant artifact in CT or poor opacification in CT or ICA that could conceal a ≥ 50% stenosis in a vessel with a reference diameter of ≥ 2 mm without an obstructive coronary artery stenosis elsewhere in the same patient. Participants with nondiagnostic initial tests were recommended to undergo further testing

### CT analysis

CT examinations were performed at certified clinical centers with ≥ 64-slice technology using either retrospectively ECG-gated or prospectively ECG-triggered scan modes as listed in the main trial results publication [15]. All examinations were performed and reported by at least level-II-certified specialists with at least 5 years of experience at each clinical center according to a 10-step imaging guide and with CT scanner-specific standardized scan parameter recommendations [9]. At least one CT reader per center was required to have level-III certification for cardiac CT. All coronary CT examinations were evaluated locally by a single certified reader per patient. Coronary artery calcium scoring was performed using the specific software, depending on local availability and site preference. In participants randomized to an initial CT-guided patient management, a specific standard operating procedure (SOP) including details on plaque assessment was implemented (Supplementary Fig. 1). CAC scoring was performed using the Agatston method to calculate the total calcium burden across all coronary arteries.

Stenosis location, degree of stenosis severity and HRP features were recorded along with imaging parameters, image quality, and results from CAC scoring by the study sites. A patient was defined as having HRP features when any of the following features were present: low attenuation plaque (LAP), positive remodeling, napkin-ring sign or a total CAC score ≥ 400 Agatston units (AU) (Supplementary Figs. 1, 2). We included high CAC scores in the combined HRP definition because they have been shown to influence the accuracy of detecting traditional HRP features on cardiac CT, and, as an established risk factor for MACE, they commonly prompt risk factor modification and preventive therapy. [1–4]. LAP was defined based on the vessel cross-section as an average region of interest (ROI) attenuation ≤ 50 Hounsfield units (HU) based on intravascular imaging studies [9, 16, 17]. Positive remodeling was defined using a remodeling index threshold of ≥ 1.1 [1, 18, 19]. The napkin-ring sign was defined on the vessel cross-section as a plaque with a central area of low attenuation in contact with the lumen, surrounded by a ring with higher attenuation [20]. A total CAC score ≥ 400 AU was also considered a high-risk feature reflecting the total coronary plaque burden and its association with a high risk of subsequent MACE in symptomatic patients [21]. The presence of 50% stenosis or greater in any vessel was defined as obstructive CAD.

Participants were divided into 4 CT plaque categories based on the presence of obstructive CAD or the combined HRP definition:

- obstructive CAD (≥ 50%) or nondiagnostic image quality
- obstructive CAD (≥ 50%) or nondiagnostic image quality and presence of HRP
- no obstructive CAD (< 50%) and HRP present
- no obstructive CAD (< 50%) and no HRP present, including participants with normal coronary arteries.

### Participant management based on CT

The DISCHARGE CT-guided treatment strategy protocol was implemented for further management in participants who were assigned to the CT arm in the trial [9]. Participants without obstructive CAD were discharged with recommendations on guideline-oriented prevention therapy, lifestyle changes, and target values for blood pressure, serum lipids and glucose levels [9, 22]. When obstructive CAD was detected on CT, ICA or noninvasive functional testing was performed. Direct referral for ICA after CT was requested only in participants with high-risk anatomy (left main stenosis or equivalent, proximal LAD (left anterior descending) stenosis, or 3-vessel disease), whereas, in participants with 1- or 2-vessel disease, the local heart team decided which locally available functional test to utilize and only referred participants if ischemia was present (ischemic burden > 10%) (Supplementary Fig. 3) [22].

The initiation of medical therapy and risk factor modification was suggested based on the DISCHARGE trial HRP definitions. Importantly, alongside traditional HRP features on CT, OMT recommendations were also given if CT demonstrated a total CAC score ≥ 400 AU (Supplementary Fig. 1), because strong evidence supports the use of CAC score ≥ 400 as a threshold to identify individuals at high risk for adverse events. These features were included because they trigger subsequent secondary prevention therapy. Additionally, we performed an exploratory analysis comparing HRP features with CAC categories to further assess their relative prognostic value (Supplementary Table 10) [21, 23]. In keeping with the pragmatic trial design, study site interpretations of plaque features were used to instigate treatment (Supplementary Fig. 3), and therapeutic decisions were made by the local heart teams [22, 24, 25].

### Endpoint definition

Follow-up visits were performed at 1 year and a median of 3.5 years. The definition of MACE as the primary outcome of this study included CV death, nonfatal myocardial infarction or stroke. The secondary endpoint was an expanded MACE composite including CV death, nonfatal myocardial infarction, nonfatal stroke, TIA, or major procedure-related complication occurring during or within 48 h after CT or ICA or related tests. Major procedure-related complications were defined as death, nonfatal myocardial infarction, nonfatal stroke, further complications prolonging hospitalization by at least 24 h, such as dissection (coronary, aorta), cardiogenic shock, cardiac tamponade, retroperitoneal bleeding, cardiac arrhythmia (ventricular tachycardia, or ventricular fibrillation) or cardiac arrest. All possible events were adjudicated by members of an independent clinical events committee, blinded to group assignment (Supplementary Methods) [14, 26].

### Statistical analysis

Categorical variables were compared using Chi-square tests (or Fisher’s exact test for small datasets). Ordinal variables were compared using a linear-by-linear association test. Continuous variables were compared between groups using analysis of variance for normally distributed data or the Kruskal–Wallis test for nonnormally distributed data. Normal distribution was assumed based on the histogram, Q-Q plot, box plot and skewness of the data.

Survival analysis using Kaplan–Meier curves was performed to compare outcomes between the 4 subgroups based on the presence of our combined HRP definition or obstructive CAD. Secondary time-to-event endpoints, such as the composite of MACE and major procedure-related complications, were also analyzed using cumulative incidences adjusting for competing risks [27]. As a sensitivity analysis, a multivariate subdistribution-Cox proportional hazard model was used to evaluate adjusted differences in the hazard risk of MACE and secondary endpoints. A sensitivity analysis was performed, excluding patients with nondiagnostic scans, applying the same set of predictors for MACE. The proportional hazard assumption of the Cox model was evaluated using “log-log” plot curves and Schoenfeld residuals. Performance of the models developed was assessed using Harrell’s C-index. A C statistic below 0.70 was considered to indicate poor discrimination.

Statistical analyses were performed using SAS software version 9.4 (SAS Institute Inc.), SPSS for Windows version 26 (IBM), and the programming language *R* version 4.0.3. In the current secondary analysis, statistical significance was defined as a two-sided *p*-value of less than 0.05, in contrast to the primary analysis, for which a *p*-value of 0.048 was considered significant because of a prespecified interim analysis of the primary outcome. No adjustment for multiple testing was performed. All analyses were performed on the intention-to-treat population.

## Results

A total of 1745 participants (mean age of 60.2 ± 10.2 years, 990/1745 female vs 755/1745 male, mean body mass index of 28.9 ± 5.1 kg/m^2^) who underwent CT and had complete data on HRP and obstructive CAD were assessed in this substudy. The mean effective radiation dose was 7.1 ± 5.1 mSv. A total of 1734/1745 (99.4%) participants completed primary outcome analysis.

### Characteristics of coronary artery disease on CT

CT showed HRP and obstructive CAD in 330/1745 (18.9%) participants, obstructive CAD alone in 211/1745 (12.1%) participants, combined HRP criteria only in 147/1745 (8.4%) participants, and neither HRP nor obstructive CAD in 1057/1745 (60.6%) participants. Participants with HRP features alone were older (mean age of 62.2 ± 9.6 vs 58.5 ± 10.3 years, *p* < 0.001) and had substantially higher pretest probability of CAD (mean 39.1 ± 10.3 vs 35.2 ± 10.6, *p* < 0.001) than those without HRP.

Age, gender, pretest probability, medication, hypertension, diabetes mellitus, and hyperlipidemia significantly differed between plaque subgroups. Details of baseline characteristics stratified by the combined HRP criteria and obstructive CAD are compiled in Table 1, whereas diagnostic and treatment strategies and coronary findings are summarized in Table 2.

### Association of HRP features and obstructive CAD with MACE

**Fig. 2:**
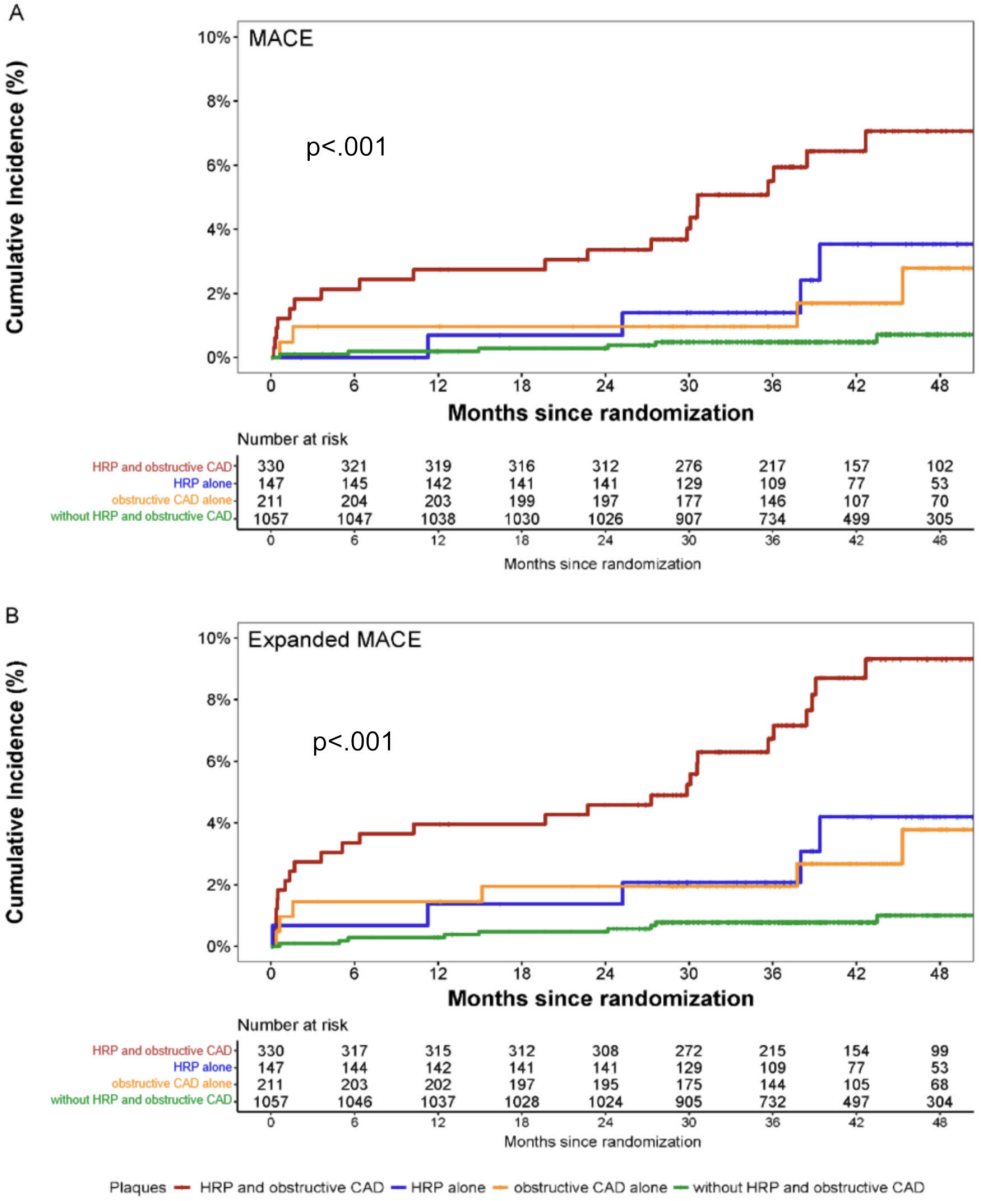
Time-to-event curves for MACE and expanded MACE stratified by HRP features and obstructive CAD on CT. Time-to-event curves for (**A**) primary endpoint (MACE) and (**B**) secondary composite endpoints of expanded MACE (cardiovascular death, myocardial infarction, stroke, transient ischemic attack, or major procedure-related complication). At a median follow-up of 3.5 years, the presence of combined HRP features and obstructive CAD resulted in significantly better prediction of MACE (HR: 8.78; 95% CI: 2.90–26.6; *p* < 0.001) and the expanded MACE composite (HR: 7.31; 95% CI: 3.06–17.48; *p* < 0.001) compared with participants without HRP or obstructive CAD. MACE, major adverse cardiovascular events; CAD, coronary artery disease; HRP, high-risk plaque

At a median follow-up of 3.5 years (IQR: 2.9–4.2), a total of 35 MACE and 47 expanded MACE events occurred. MACE included CV death (7 participants), nonfatal myocardial infarction (21 participants), and nonfatal stroke (9 participants). Time-to-event curves are presented in Fig. 2.

Participants with HRP features (147/1745) had a higher risk of MACE and expanded MACE than those without HRP or obstructive CAD (unadjusted HR: 3.90; 95% CI: 1.14–13.3; *p* < 0.01) and (unadjusted HR: 3.46; 95% CI: 1.18–10.1; *p* = 0.02), respectively. The association of the combined HRP definition with the primary and secondary endpoints persisted after controlling for risk factors including age, gender, body mass index (BMI), diabetes mellitus, hypertension, hyperlidaemia and smoking (adjusted HR: 3.81, 95% CI: 1.01–14.6, *p* = 0.050; and HR: 3.21, 95% CI: 1.07–9.66, *p* = 0.038, respectively). The combination of HRP features and obstructive CAD conferred the highest risk for both MACE (adjusted HR: 8.78; 95% CI: 2.90–26.6; *p* < 0.001) and the expanded MACE composite (adjusted HR: 7.31; 95% CI: 3.06–17.48; *p* < 0.001) compared with participants without HRP or obstructive CAD. Results for primary and secondary endpoints stratified by the combined HRP criteria and the presence of obstructive CAD are shown in Table 3 and Supplementary Table 2. In an exploratory multivariable model (Supplementary Table 10), a CAC score ≥ 400 emerged as a stronger predictor of MACE than CT-defined HRP features. In the sensitivity analysis excluding nondiagnostic scans, patients with both HRP features and obstructive CAD had the highest risk of MACE (HR: 9.44, 95% CI: 2.99–29.7, *p* < 0.001). HRP alone was also significantly associated with MACE (HR: 3.98, 95% CI: 1.02–15.5, *p* = 0.047), whereas obstructive CAD alone showed similar effect sizes but did not reach statistical significance (HR: 3.99, 95% CI: 0.90–17.6, *p* = 0.068) compared with those without HRP or obstructive CAD (Supplementary Table 11). Cumulative incidence of events is shown in Supplementary Tables 3–5.

**Table 3:** Multivariable analysis of the primary and secondary endpoints (*n* = 1745)

	HR	95% CI	*p*-value
MACE			
Without HRP and obstructive CAD	1		
Obstructive CAD alone	3.53	0.99–12.7	0.053
Combined HRP criteria only	3.81	1.01–14.6	0.050
With HRP and obstructive CAD	8.78	2.90–26.6	< 0.001
Expanded MACE			
Without HRP and obstructive CAD	1	1.24–9.67	
Obstructive CAD alone	3.47	1.07–9.66	0.017
Combined HRP criteria only	3.21	3.06–17.48	0.038
With HRP and obstructive CAD	7.31	1.24–9.67	< 0.001

Multivariable Cox proportional hazard models for MACE and expanded MACE composite. Both models were adjusted by age, gender, BMI, diabetes mellitus, hypertension, hyperlipidemia and smoking

*CAD* coronary artery disease, *HRP* high-risk plaque, *MACE* major adverse cardiovascular events

Taking plaque categories into account in addition to risk factors improved prognostication of MACE and expanded MACE composite (model with risk factors: Harrell’s C 0.701 [0.635–0.768] vs model with risk factors and plaque categories: 0.752 [0.695–0.809] for MACE and 0.699 [0.639–0.759] vs 0.745 [0.694–0.796] for expanded MACE, respectively) (Supplementary Table 6).

### Secondary prevention therapy after CT imaging

At a median of 3.5 years, statin therapy was administered in 349/957 participants (33.0%) without obstructive CAD or HRP, in 67/121 participants (45.6%) with HRP alone, in 129/189 participants (61.1%) with obstructive CAD alone, and in 222/289 (67.3%) with both obstructive CAD and HRP. Comparison of baseline and follow-up data on statin administration revealed that statin was initiated after CT imaging in 123/1057 (11.6%) participants without obstructive CAD or HRP, in 28/147 (19.1%) participants with HRP alone, in 48/211 (22.8%) participants with obstructive CAD alone, and in 88/330 (26.7%) with both HRP feature and obstructive CAD. Further data on secondary prevention therapies after the initial CT are summarized in Supplementary Tables 7, 8.

## Discussion

The aim of this prespecified analysis of the DISCHARGE trial was to investigate the prognostic value of HRP features, combining traditional HRP features with a high coronary artery calcium score for adverse events. In low-to-intermediate risk participants with stable chest pain initially referred to invasive coronary angiography, our main finding was that the presence of HRP features was strongly associated with MACE and expanded MACE composite in participants without obstructive coronary artery disease compared to those without any HRP. HRP features alone without obstructive CAD were as predictive of MACE as obstructive CAD alone without HRP features. The presence of HRP improved risk assessment even after adjusting for risk factors for MACE (3.8-fold increased risk) and expanded MACE composite (3.2-fold increased risk). Adding HRP information to the prediction models improved their discriminatory power for events. Therefore, the implementation of plaque features has the potential to optimize patient management by identifying patients who benefit from a more aggressive prevention therapy and a more relentless control of other CV risk factors.

### CT-based assessment of high-risk plaque features

Histopathological investigations indicate that the majority of acute events originate from the sudden rupture of a “vulnerable” plaque with subsequent coronary artery thrombosis [20, 28]. Certain plaque characteristics are well-known markers of plaque vulnerability from intravascular ultrasound (IVUS) and optical coherence tomography studies, including the thin fibrous cap surrounding a lipid-rich, large necrotic core or intraplaque microcalcifications and hemorrhage [29, 30]. While most of these markers are not directly visible with CT technology due to its lower spatial resolution, CT resolution is sufficient for accurate visualization of the vessel wall and plaque morphology, allowing the establishment of CT surrogates of HRP [20]. Most single-center, observational CTA trials investigating HRP features in patients with stable chest pain have found a strong association with MACE for positive remodeling, low plaque attenuation and the napkin-ring [12, 28, 31]. Key CT trials on the prognostic value of HRP features are described in Supplementary Table 9. Furthermore, HRP features were significantly associated with acute coronary syndrome in patients presenting to the emergency department with an initial negative ECG and troponin, regardless of the presence of obstructive CAD and clinical risk factors [32]. Similar to the PROMISE trial, we did not include spotty calcification in our definition since meta-analyses have shown that among the traditional HRP features, spotty calcification has the lowest prognostic value [33]. Inter-reader variability is a well-recognized limitation of CT-based assessment of HRP features. Because the napkin-ring sign is a qualitative marker, its identification is dependent on reader experience and subject to inter-observer variability. Similarly, LAP assessment relies on different HU thresholds in the literature and is prone to variability [11, 19].

### Prognostic value of HRP features

Published data on the prognostic value of HRP features are influenced by the definition of plaque vulnerability, and there are substantial differences across investigations [8, 12, 13]. The SCOT-HEART trial investigated the prognostic value of plaque characterization by CTA, including positive remodeling and LAP < 30 HU, for the prediction of CAD-related mortality or nonfatal myocardial infarction [13]. In 1769 participants with stable symptoms who were followed up for 5 years, the highest risk for events was found in participants with both obstructive disease and HRP features (HR: 11.50, 95% CI: 3.39–39.04; *p* < 0.001) as compared with participants with normal coronaries, although the relationship was not independent of the CAC score. Our study found similar results where participants with both obstructive CAD and HRP features had the highest event rate with a cumulative incidence of 7.1% for MACE and 9.3% for expanded MACE, respectively. A later substudy of the SCOT-HEART trial [34], however, showed that quantitatively assessed lipid-rich low-attenuation plaque with a CT number of less than 30 HU was the strongest predictor of myocardial infarction (adjusted HR: 1.60, 95% CI: 1.10–2.34 per doubling; *p* = 0.014), irrespective of CV risk factors, CAC score, or area stenosis. Patients with a LAP burden greater than 4% were nearly 5 times more likely to have a subsequent myocardial infarction (HR: 4.65, 95% CI: 2.06–10.5; *p* < 0.001).

The nested substudy of the Prospective Multicenter Imaging Study for Evaluation of Chest Pain (PROMISE) evaluated 4415 stable patients who were assigned to the CT arm (initial anatomical strategy) [12]. The predictive value of HRP features (having either positive remodeling, LAP, or the napkin-ring sign) was independent of CV risk factors and obstructive CAD for detecting MACE. Our results corroborate the observation that HRP features, including the napkin-ring sign, LAP, positive remodeling, and a CAC score ≥ 400 AU, confer a higher risk for MACE in participants with and without obstructive CAD. Moreover, HRP features without obstructive CAD were as predictive of MACE as obstructive CAD alone without coronary HRP features, demonstrating the clinical value of including such features in risk assessment. A CAC score ≥ 400 AU as a high-risk feature indicates advanced CAD and is a strong predictor of CV events in symptomatic patients. Performing CAC scoring before CCTA and including it in the diagnostic model enhances the accuracy of diagnosing obstructive CAD, especially in cases where CCTA alone is nondiagnostic. This was included to ensure that patients received recommendations for preventive measures based on individual risk. The DISCHARGE trial consistently showed a strong association with adverse events, and we suggest that this definition of HRP could also be implemented in updated treatment recommendations.

Although the presence of both obstructive CAD and HRP features was associated with a high risk of MACE with a hazard ratio of 8.78 (95% CI: 2.90–26.6), this was lower than the hazard reported in the SCOT-HEART (HR: 11.5, 95% CI: 3.39–39.04) or PROMISE (HR: 12.24, 95% CI: 6.22–24.1) trials. This might be attributable to the different MACE rates and the changes in optimal medical therapy and the intensification of risk factor management triggered by the identification of HRP features according to the DISCHARGE protocol. The expanded MACE composite also occurred more frequently in participants with HRP or both HRP and obstructive CAD, likely explained by the known increased risk of periprocedural complications from more extensive calcifications encountered during ICA, more complex and advanced stages of CAD, and a higher coronary plaque burden [35].

### Secondary prevention therapy

Recently, diagnostic criteria of HRP and management decisions based on plaque assessment by CTA have been implemented in guidelines and expert consensus documents [5, 36]. Notably, the DISCHARGE trial incorporated recommendations on patient management, including optimal medical therapy and CV risk factor modification, while medications were controlled and adjusted at follow-up visits to ensure adherence to therapy (e.g., statin, antiplatelet medication) [6, 22, 37, 38]. Patients with either HRP or obstructive CAD may benefit from aggressive secondary prevention, including lipid-lowering and risk factor control. Those with both HRP and obstructive CAD had the highest risk and may warrant even lower LDL targets and intensified therapy.

A large proportion of adverse events occurred in participants without an obstructive lesion, and HRP features were utilized to refine risk assessment and guide management. Approximately one quarter of participants with HRP alone (26.5%) were already on statins before enrollment, yet a substantial proportion of additional participants (19.1%) started statin therapy following CT. A large number of participants (40%) with both obstructive CAD and the combined HRP definition were already taking statin therapy at enrollment, yet additional participants (26.7%) commenced statin therapy based on the CT findings (Supplementary Tables 7, 8). Although not analyzed in the DISCHARGE trial, it is worth noting that CT-based education intervention can increase statin prescription and improve adherence to lipid-lowering therapy, as shown in a subanalysis of the CAD-Man trial (NCT00844220) [26, 39]. Our findings suggest potential opportunities for enhancing preventive efforts, including statin therapy.

### Study limitations

This substudy has several limitations. The MACE and expanded MACE rates observed are comparable with other recent trials but are relatively low in absolute terms, which limits the generalizability of our conclusions. The numbers of patients in the obstructive CAD alone (*n* = 211), combined HRP criteria only (*n* = 147), and HRP and obstructive CAD (*n* = 330) subgroups were relatively small compared to the group without obstructive CAD or HRP features (*n* = 1057). The DISCHARGE trial only included patients with a pretest probability of < 60%, excluding higher-risk patients, who might be more likely to have HRP features. Furthermore, quantitative plaque assessment was not performed and segment involvement metrics were not analyzed for event prediction. We used a combined definition of HRP—including CACS ≥ 400 and traditional plaque features—as predefined in the protocol, since these findings typically trigger risk factor modification and preventive therapy in clinical practice, although this approach may limit the ability to assess their individual contributions to MACE. Nevertheless, these features represent different CAD phenotypes vulnerability and burden, and were grouped together for pragmatic reasons. While traditional HRP features capture local plaque vulnerability, the overall atherosclerotic burden reflected by CAC ≥ 400 may carry greater prognostic weight in predicting adverse outcomes. The use of site-specific calcium scoring software may have introduced variability in CAC quantification across centers, which could have affected the consistency of the results. Moreover, inter-reader variability in identifying HRP features cannot be excluded. In our analysis, we included patients with nondiagnostic coronary CT scans to preserve the integrity of the cohort and reflect real-world clinical practice, where non-evaluable segments due to motion artifacts, heavy calcification, or poor contrast opacification are not uncommon. According to the study protocol, any coronary segment affected by artifacts that could conceal a ≥ 50% stenosis in a revascularization-eligible vessel (≥ 2 mm reference diameter) must be considered positive for obstructive CAD for management purposes. Although we applied a ≤ 50 HU threshold for LAP in accordance with the predefined analysis plan and prior IVUS–CTA studies, we acknowledge that alternative cut-offs, particularly < 30 HU, have also been associated with higher risk and may lead to different stratification.

## Conclusions

High-risk plaque features that included traditional features with high coronary artery calcium scores on CT were strongly associated with major adverse cardiovascular events and expanded major adverse cardiovascular event composite in participants without obstructive coronary artery disease. High-risk plaque features on coronary CT provide independent and incremental predictive value for major adverse cardiovascular events and expanded major adverse cardiovascular event composite after adjusting for cardiovascular risk factors in a large pan-European trial. Coronary high-risk plaque features alone without obstructive coronary artery disease were as predictive of major adverse cardiovascular events as obstructive coronary artery disease alone without coronary high-risk plaque features.

## Supplementary information

ELECTRONIC SUPPLEMENTARY MATERIAL

## Data Availability

Data collected for our study will be made available to others in the form of partial datasets, including individual participant data that underlie the results reported in this article after deidentification (text, tables, figures and appendices). Study center individual patient data will not be disclosed because of the possibility of re-identification of patients. Researchers who wish to access data should send an email to discharge.eu@charite.de. To gain access, requestors will have to sign a data sharing agreement. Data availability will begin 12 months after publication and end 3 years after publication. Data will be shared with researchers who provide a methodologically sound proposal approved by the Dissemination Committee of the DISCHARGE trial. The study proposal must include: The proposed study’s overview, rationale, aims and analysis methods, plans for dissemination of results and names of those wishing to access data, and how the data are going to be stored and for how long. Proposals must not infringe the rights of the DISCHARGE investigators to perform their predefined primary and secondary outcomes analyses and publications as laid out in the Consortium Agreement with the European Commission and listed at https://clinicaltrials.gov/ct2/show/NCT02400229 or the Statistical Analysis Plan. Data will principally be made available for individual participant data for systematic reviews and meta-analysis. To be eligible for a request, IPD meta-analyses need to be registered on PROSPERO, have a study protocol that follows the PRISMA-P guideline and checklist, and will publish their results following the PRISMA-IPD statement. If the proposal is for collaborative analysis of data from several trials, the DISCHARGE trialists request to be considered as authors based on the proportion of patients provided for such an analysis and according to ICMJE authorship criteria.

## Abbreviations

CAC: Coronary artery calcium
CAD: Coronary artery disease
DISCHARGE: Diagnostic Imaging Strategies for Patients with Stable Chest Pain and Intermediate Risk of Coronary Artery Disease
HR: Hazard ratio
HRP: High-risk plaque
LAP: Low CT attenuation plaque
PROMISE: Prospective Multicenter Imaging Study for Evaluation of Chest Pain
SCOT-HEART: The Scottish Computed Tomography of the HEART
TIA: Transient ischemic attack
