# Supplementary material for: Association of high-risk CT coronary artery plaque features with major adverse cardiovascular events: a prespecified secondary analysis of the DISCHARGE trial
Source: Eur Radiol. 2026 Feb 13;36(7):5502–17. doi: 10.1007/s00330-025-12146-3 (PMC13282328; doi:10.1007/s00330-025-12146-3)
Supplement: Supplementary file 1 — ELECTRONIC SUPPLEMENTARY MATERIAL [file 330_2025_12146_MOESM1_ESM.pdf]

Association of High-Risk CT Coronary Artery Plaque Features with Major Adverse Cardiovascular Events: A Prespecified Secondary Analysis of the DISCHARGE Trial

ELECTRONIC SUPPLEMENTARY MATERIAL

Randomization

A web-based system (SecuTrial ®) was used for randomly assigning participants (1:1) to either ICA or CT as the initial examination after confirming eligibility criteria and recoding data for individual calculation of pretest probability of disease. Block randomization applied computer-generated and randomly permuted blocks with a length of 4, 6 or 8, stratified according to center, with central assignment. Randomization was stratified by gender at each clinical center.

Power calculation

A post hoc power calculation was conducted to estimate the minimum effect size of the main effects for “plaques” required to achieve a power of 80% at 5% alpha level. The post hoc power calculation was performed for the primary outcome MACE and was based on the Cox proportional hazard regression test accounting for competing risk. When the sample size is 1745, with a total number of the first co-primary endpoint (MACE) of 90, an exponential maximum likelihood test of equality of survival curves with a 0.05 two-sided significance level (α) will have 80% power to detect the difference between an exponential parameter, $\lambda 1$ of 0.0016 (three-year MACE incidence equal to 0.48%) and an exponential parameter, $\lambda 2$, of 0.0046 (three-year MACE incidence equal 1.38%) and thus a constant hazard ratio of 2.89. The cumulative incidence proportion for the competing risk factors at 3 years was assumed to be 2% in both trial arms. Furthermore, the sample size estimation assumes an accrual period of 3.5 years and a conservatively estimated common exponential dropout rate of 0.031 (0.31% per year) as the loss for MACE in the DISCHARGE trial was only 1.1% after 3.5 years.

CT analysis in the trial

CT examinations were performed at certified clinical centers with ≥64-slice scanner technology. The annual average number of all cardiac CT procedures per center was a mean of 1213 (min: 120, max: 6800). CT images were acquired according to the “10–steps guide to performing cardiac CT” and scanner-specific guides developed by the DISCHARGE consortium. To ensure uniform, standardized and proper reporting of coronary CT findings, hands-on CT trainings were organized by board-certified experts, and a standard operating procedure (SOP) on CT-based plaque characterization was created. All CT images were sent to a central core lab as DICOM images, while the reports from the clinical centers were entered in the electronic case report forms. It was recommended to use double oblique views, multi-planar reformations, and cross-sections in all coronary artery segments. Diameter stenosis was recorded based on proximal and distal reference measurements and lesion location was added based on the 18 segment AHA-classification scheme.

Nondiagnostic image quality was defined as a relevant artifact in CT or poor opacification in CT or ICA that could conceal a ≥50% stenosis in a vessel with a reference diameter of ≥2 mm without obstructive stenosis elsewhere. Participants with nondiagnostic initial test results were recommended to undergo further testing.

We did not include spotty calcification in our HRP definition since meta-analyses have shown that among HRP features spotty calcification has the lowest prognostic value with some data even suggesting that it has no effect at all. While NRS has probably the lowest reproducibility among experts, spotty calcium is also highly variable.

Low attenuation plaque definition

In our study, LAP was defined as an average region of interest (ROI) attenuation of ≤50 HU on the cross-sectional images.[1] The differentiation of lipid-rich and fibrous plaques based on CT attenuation is unreliable, as comparative studies with IVUS have found substantial overlap in CT density.[2] Therefore, Kristanto et al. proposed that LAP should be allowed to have >50 HU if the adjacent lumen has a high attenuation.[3] Importantly, lower CT attenuation (<30 HU) correlates with a higher lipid-necrotic core plaque component and confers the highest risk, while lesions with <60 HU have also been reported to be powerful prognosticators of events.[4] Despite the recent consensus document advocating for a 30 HU cutoff value for LAP[5], our analysis relied on a predefined SAP that employed a 50 HU threshold utilized in studies combining IVUS and CTA.[6]

Endpoint definition

Participants were enrolled at 26 centers in 16 European countries. The definition of MACE included cardiovascular death, nonfatal myocardial infarction or stroke. Only symptomatic events were defined as MACE according to the study protocol. The standardized definitions for cardiovascular and stroke end point events in clinical trials by the Cardiac Safety Research Consortium were implemented[7]. In accordance, cardiovascular death included death resulting from acute myocardial infarction, sudden cardiac death, death due to heart failure, death due to stroke, death due to cardiovascular procedures, death due to cardiovascular haemorrhage or death due to other cardiovascular causes.

The follow-up period lasted from randomization until the occurrence of the outcome, or otherwise censored at death (noncardiovascular events and unknown causes of death), lost to follow-up or end of the study.

The third universal definition of myocardial infarction (MI) of the ESC/ACCF/AHA/WHF Task Force was implemented to define non-fatal MI at the study period of current trial.[8] An event was categorized as nonfatal if it did not result in the patient's death. Cardiac troponin I or T (cTn) was the preferred biomarker for diagnosis with creatine kinase MB isoform (CKMB) as the best alternative option.

The definition of stroke by the AHA/ASA was applied.[9] Ischemic stroke was characterized as a neurological dysfunction episode originating from localized infarction within the central nervous system (CNS). Nonfatal stroke was classified, as indicated by the AHA/ASA Task Force, into the following categories: hemorrhagic infarction, cerebral hemorrhage (intracerebral hemorrhage, subarachnoid hemorrhage, intraventricular hemorrhage), and cerebral venous thrombosis.

The list of major procedure-related complications followed the definitions of the CAD-Man trial[10] and was predefined in the study protocol as follows:

- Death
- Nonfatal myocardial infarction
- Nonfatal stroke
- Further complications prolonging hospitalization by at least 24 hours
- Dissection (coronary, aorta)
- Cardiogenic shock
- Cardiac tamponade
- Retroperitoneal bleeding
- Cardiac arrhythmia (ventricular tachycardia, ventricular fibrillation)
- Cardiac arrest

References:

1. Yamaki T, Kawasaki M, Jang I-K, et al. Comparison between integrated backscatter intravascular ultrasound and 64-slice multi-detector row computed tomography for tissue characterization and volumetric assessment of coronary plaques. Cardiovascular Ultrasound. 2012;10(1):33.
2. Leber AW, Knez A, Becker A, et al. Accuracy of multidetector spiral computed tomography in identifying and differentiating the composition of coronary atherosclerotic plaques: a comparative study with intracoronary ultrasound. Journal of the American College of Cardiology. 2004;43(7):1241-7.
3. Kristanto W, van Ooijen PM, Jansen-van der Weide MC, Vliegenthart R, Oudkerk M. A meta analysis and hierarchical classification of HU-based atherosclerotic plaque characterization criteria. PLoS One. 2013;8(9):e73460.
4. Maurovich-Horvat P, Schlett CL, Alkadhi H, et al. The napkin-ring sign indicates advanced atherosclerotic lesions in coronary CT angiography. JACC Cardiovasc Imaging. 2012;5(12):1243-52.
5. Narula J, Chandrashekhar Y, Ahmadi A, et al. SCCT 2021 Expert Consensus Document on Coronary Computed Tomographic Angiography: A Report of the Society of Cardiovascular Computed Tomography. J Cardiovasc Comput Tomogr. 2021;15(3):192-217.
6. Uetani T, Amano T, Kunimura A, et al. The Association Between Plaque Characterization by CT Angiography and Post-Procedural Myocardial Infarction in Patients With Elective Stent Implantation. JACC: Cardiovascular Imaging. 2010;3(1):19-28.
7. Hicks KA, Tcheng JE, Bozkurt B, et al. 2014 ACC/AHA Key Data Elements and Definitions for Cardiovascular Endpoint Events in Clinical Trials: A Report of the American College of Cardiology/American Heart Association Task Force on Clinical Data Standards (Writing Committee to Develop Cardiovascular Endpoints Data

Standards). J Am Coll Cardiol. 2015 Jul 28;66(4):403-69. doi: 10.1016/j.jacc.2014.12.018. Epub 2014 Dec 29.

8. Thygesen K, Alpert JS, Jaffe AS, et al. Third universal definition of myocardial infarction. Eur Heart J. 2012 Oct;33(20):2551-67. doi: 10.1093/eurheartj/ehs184. Epub 2012 Aug 24. PMID: 22922414.
9. Sacco RL, Kasner SE, Broderick JP, Caplan LR, Connors JJ, Culebras A, Elkind MS, George MG, Hamdan AD, Higashida RT, Hoh BL, et al. An updated definition of stroke for the 21st century: a statement for healthcare professionals from the American Heart Association/American Stroke Association. Stroke. 2013 Jul;44(7):2064-89. doi: 10.1161/STR.0b013e318296aeca. Epub 2013 May 7.
10. Dewey M, Rief M, Martus P, et al. Evaluation of computed tomography in patients with
atypical angina or chest pain clinically referred for invasive coronary angiography: randomised
controlled trial. BMJ 2016;355:i5441. doi:10.1136/bmj.i5441

Supplementary Tables

Supplementary Table 1. DISCHARGE Trial Patient Representativeness (total patient population of the DISCHARGE trial)

Disease, problem, or condition under investigation	Participants referred for invasive coronary angiography (ICA) to 26 European centers with stable chest pain and 10-60% probability of CAD.
Special considerations related to Gender	56% of participants were female.
Age	Participants younger than 30 years were excluded from the study. There was no upper age limit for inclusion. 62% of participants were 45-65 years old, 31% were older than 65 years, and 8% of participants were younger than 45 years.
Ethnicity	The study population was typical for a European patient cohort with 99% being Caucasian, 0.3% Asian, 0.2% Indian, and 0.1% Black.
Countries	Participants were from 16 countries and included all European regions (North: Denmark, Latvia, Finland; Central: Germany, Austria; East: Czech Republic, Hungary, Lithuania, Poland, Romania, Serbia; South: Italy, Portugal, Spain; West: United Kingdom, Ireland)
Continental region	44% of participants were from Eastern Europe, followed by 20% of participants from Northern Europe, 14% of participants from Western Europe, 13% of participants from Southern Europe, and 8% of participants from Central Europe.
Overall representativeness of this trial	The study is representative of participants referred for ICA with stable chest pain and intermediate pretest probability of CAD (10-60%)
Income level	Patients included in the study cohort reported a personal monthly income of 623.6 (300.0–1504.2) Euros.
Marital status	67% of participants were married or in a registered partnership, 14% were divorced or reported a registered partnership that was legally dissolved, 11% were widowed or with registered partnership that ended with death of partner, 8% were never married and never in a registered partnership.
Employment status	41% of participants were employed, 40% of participants were retired, 5% of participants were unemployed, 4% of participants fulfilled domestic tasks, and 2% of participants reported to be permanently disabled.

The DISCHARGE consortium, a multinational European research group, is conducting the DISCHARGE trial with a patient population that displays a wide range of characteristics. These variations arise from the diverse geographical and economic conditions prevalent throughout Europe.

Supplementary Table 2. Primary and secondary endpoints based on the presence of HRP features or obstructive CAD on CT (n = 1,745)

CAD= coronary artery disease, HRP= high-risk plaque, MACE= major adverse cardiovascular events

	**Without HRP and obstructive CAD (n=1057)**		**Obstructive CAD alone (n=211)**			**Combined HRP criteria only (n=147)**			**With HRP and obstructive CAD (n=330)**		
	**event (%)**	**HR (95% CI)**	**event (%)**	**HR (95% CI)**	**P-value**	**event (%)**	**HR (95% CI)**	**P-value**	**event (%)**	**HR (95% CI)**	**P-value**
Major adverse cardiovascular events (MACE)	7 (0.6%)	1	4 (1.9%)	2.91 (0.86 - 9.82)	0.09	4 (2.7%)	3.90 (1.14 - 13.3)	<0.01	20 (6.1%)	9.32 (3.93 - 22.1)	<0.001
Nonfatal myocardial infarction	5 (0.5%)	1	2 (0.9%)	2.05 (0.40 - 10.3)	0.39	3 (2.0%)	4.11 (0.97 - 17.4)	0.06	11 (3.3%)	7.02 (2.42 - 20.3)	<0.001
Expanded MACE composite	10 (0.9%)	1	6 (2.8%)	3.07 (1.12 - 8.40)	0.03	5 (3.4%)	3.46 (1.18 - 10.1)	0.02	26 (7.9%)	8.57 (4.13 - 17.8)	<0.001

For the composite endpoint, data are for the first event only. The table summarizes the event rates across subgroups based on CAD severity and the combined HRP definition.

Supplementary Table 3. Cumulative incidence of events in the total cohort (n =1,745)

	6 months	1 year	3 years
**MACE**	0.72%	0.89%	1.68%
**Nonfatal myocardial infarction**	0.55%	0.61%	0.97%
**Nonfatal Stroke**	0.17%	0.22%	0.54%
**Cardiovascular death**	0.0%	0.06%	0.23%
**Expanded MACE**	1.11%	1.28%	2.25%

MACE= major adverse cardiovascular events

Supplementary Table 4. Cumulative incidence of MACE across subgroups

	**6 months**	**1 year**	**2 years**	**3 years**	**4 years**
**Without HRP and obstructive CAD**	0.19%	0.19%	0.29 %	0.48%	0.71%
**Obstructive CAD**	0.97%	0.97%	0.97%	0.97%	2.75%
**Combined HRP criteria**	0.00%	0.68%	0.68%	1.37%	3.43%
**With HRP and obstructive CAD**	2.13%	2.74%	3.36%	5.50%	7.05%

CAD= coronary artery disease, HRP= high-risk plaque

Supplementary Table 5. Cumulative incidence of expanded MACE across subgroups

	**6 months**	**1 year**	**2 years**	**3 years**	**4 years**
**Without HRP and obstructive CAD**	0.28%	0.28%	0.48%	0.78%	0.99%
**Obstructive CAD**	1.45%	1.45%	1.94%	1.94%	3.74%
**Combined HRP criteria**	0.68%	1.37%	1.37%	2.06%	4.12%
**With HRP and obstructive CAD**	3.35%	3.96%	4.58%	6.72%	9.29%

CAD= coronary artery disease, HRP= high-risk plaque

Supplementary Table 6. Harrell's C-index and comparison between univariable and multivariable models

	**Harrell's C-index**	**Log likelihood Chi2 (Model 2 vs. Model 3)**
**MACE**		
Univariable Model 1	**0.686 (0.633 - 0.738)**	
Multivariable Model 2	**0.701 (0.635 – 0.768)**	
Multivariable Model 3	**0.752 (0.695 - 0.809)**	**<0.001**
**Extended MACE**		
Univariable Model 1	**0.680 (0.633 - 0.726)**	
Multivariable Model 2	**0.699 (0.639 - 0.759)**	
Multivariable Model 3	**0.745 (0.694 - 0.796)**	**<0.001**

Univariable model: 1 include plaques subgroups

Multivariable model 2: includes age, gender, BMI, diabetes and smoking

Multivariable model 3: includes age, gender, BMI, diabetes, smoking and plaque categories.

BMI= body mass index, MACE= major adverse cardiovascular events

Plaque categories were as follows: (1) obstructive CAD (≥50%) or nondiagnostic image quality, (2) obstructive CAD (≥50%) or nondiagnostic image quality and presence of HRP, (3) no obstructive CAD (<50%) and HRP present , (4) no obstructive CAD (<50%) and no HRP present, including participants with normal coronary arteries.

Supplementary Table 7. Secondary prevention therapy as recorded on follow-up of 3.5 years (IQR: 2.9-4.2).

		**Without HRP and obstructive CAD (n=957)**		**Obstructive CAD alone (n=189)**		**Combined HRP criteria only (n=121)**		**With HRP and obstructive CAD (n=289)**	
		N	%	N	%	N	%	N	%
Statin	No	608	57.5	60	28.4	54	36.7	67	20.3
	Yes	349	33.0	129	61.1	67	45.6	222	67.3
Antiplatelet agent	No	692	65.5	68	32.2	71	48.3	69	20.9
	Yes	265	25.1	121	57.3	50	34.0	220	66.7
Beta-blocker	No	610	57.7	92	43.6	52	35.4	132	40.0
	Yes	347	32.8	97	46.0	69	46.9	157	47.6
CCB	No	758	71.7	142	67.3	98	66.7	192	58.2
	Yes	199	18.8	47	22.3	23	15.6	97	29.4
ACE-I/ARB	No	521	49.3	76	36.0	51	34.7	108	32.7
	Yes	436	41.2	113	53.6	70	47.6	181	54.8

ACE-I= angiotensin-converting enzyme inhibitor, ARB= angiotensin receptor blocker, CAD= coronary artery disease, CCB= calcium channel blocker, HRP= high-risk plaque

Supplementary Table 8. Changes in statin therapy between enrollment and on follow-up at 3.5 years (IQR: 2.9-4.2).

		Without HRP and obstructive CAD		Obstructive CAD alone		HRP alone		With HRP and obstructive CAD	
		N	%	N	%	N	%	N	%
Statin	continued	223	21.1	81	38.4	39	26.5	132	40.0
	stopped	149	14.1	24	11.4	17	11.6	27	8.2
	never	455	43.1	35	16.6	37	25.2	40	12.1
	new	123	11.6	48	22.8	28	19.1	88	26.7

CAD= coronary artery disease, HRP= high-risk plaque

Supplementary Table 9. Key CT Trials on High-Risk Coronary Plaque Features and Cardiovascular Outcomes

Trial Name	Year	Study Population	High-Risk Plaque Features	Primary Outcomes	Key Findings
**SCOT-HEART**	2019	1,778 stable chest pain patients	Positive remodeling, low-attenuation plaque, spotty calcification, napkin ring sign	coronary heart disease death or nonfatal myocardial infarction	Identification of HRP features on CTA was associated with a higher rate of events.
**PROMISE**	2018	4,415 stable chest pain patients	Positive remodeling, low-attenuation plaque, napkin-ring sign	MACE (death, myocardial infarction, or unstable angina).	Presence of high-risk plaque features carried a 70% increased risk of future MACE over a median follow-up of 25 months.
**ROMICAT-II**	2014	472 acute chest pain patients with suspected ACS	Positive remodeling, low-attenuation plaque, spotty calcification, napkin ring sign	ACS (acute MI or unstable angina pectoris)	HRP was associated with ACS independently and incrementally to the presence of significant CAD and clinical risk assessment.
**Motoyama et al.**	2015	449 stable patients with known or suspected CAD	positive remodeling and/or low density plaque	ACS	HRP was an independent predictor of ACS
**Current DISCHARGE subanalysis**	2024	1,745 patients with stable chest pain	Positive remodeling, low-attenuation plaque, napkin-ring sign, total CACS ≥400 Agatston Units	MACE (CV death, nonfatal myocardial infarction or stroke)	HRP features provide independent and incremental predictive value for MACE after adjusting for cardiovascular risk factors.<br>HRP features alone without obstructive CAD were as predictive of MACE as obstructive CAD alone without HRP features.

Supplementary Table 10. Multivariable analysis using the variable HRP (defined as the presence of LAP, or positive remodeling or napkin ring) and CAC score categories for MACE prediction (exploratory analysis in 1745 patients)

**Variable**	**HR**[1]	**95% CI**[1]	**p-value**
Traditional HRP features	1.68	0.80 – 3.52	0.170
CAC Score			
0	1		
1 – 399	3.52	0.97 – 12.7	0.055
≥ 400	10.85	2.58 – 45.6	0.001

[1] HR = Hazard Ratio, CI = Confidence Interval

Adjusted by gender, age, body mass index, diabetes, smoking, hyperlipidemia, and hypertension

Supplementary Table 11. Predictors of major adverse cardiovascular events (MACE) after excluding patients with non-diagnostic coronary CT angiography (n=1647)

**Variable**	**HR[1]**	**95% CI[1]**	**p-value**
without HRP and obstructive CAD	1		
obstructive CAD alone	3.99	0.90 – 17.6	0.068
combined HRP definition alone	3.98	1.02 – 15.5	0.047
with HRP and obstructive CAD	9.44	2.99 – 29.7	<0.001

1 HR = Hazard Ratio, CI = Confidence Interval,

Adjusted by gender, age, body mass index, diabetes, smoking, hyperlipidemia, and hypertension

Supplementary Figures

Supplementary Figure 1. High-risk plaque features in the DISCHARGE trial

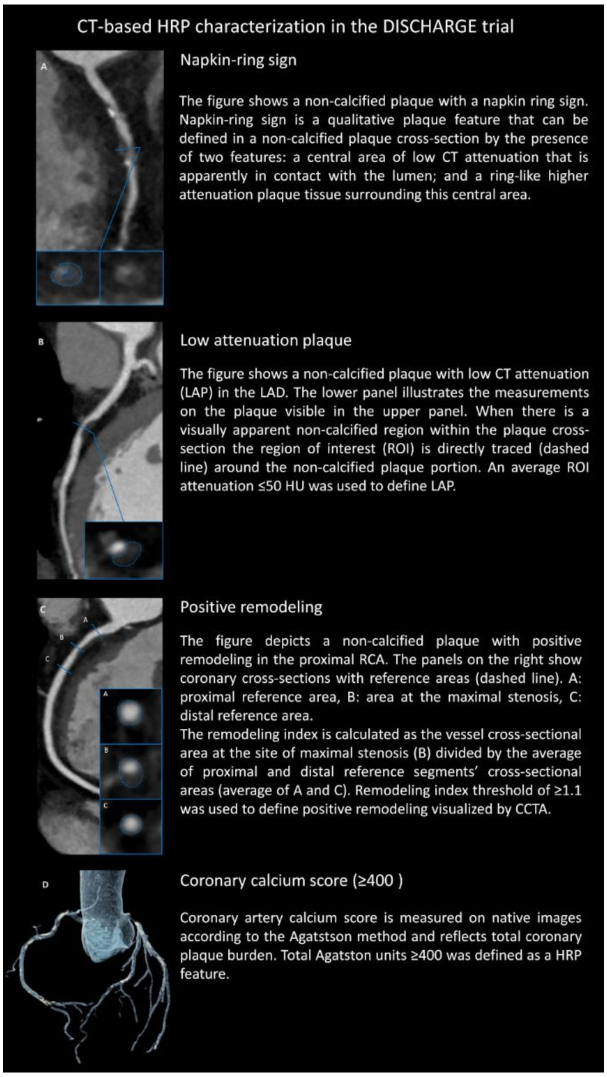

The figure summarizes the HRP features of the trial based on the predefined DISCHARGE trial protocol. Qualitative (napkin-ring sign) **(A)** and semi-quantitative (low attenuation plaque and positive remodeling) **(B & C)** features were recorded alongside a CAC score ≥400 **(D)** for each patient by the site readers using dedicated software tools available for site reads. Patients with HRP was defined by having any of these four HRP features.

HRP= High-risk plaque, LAD= Left anterior descending, LAP= Low attenuation plaque, RCA= Right coronary artery, ROI= Region of interest

Supplementary Figure 2. Representative example of a coronary lesion with HRP characteristics.

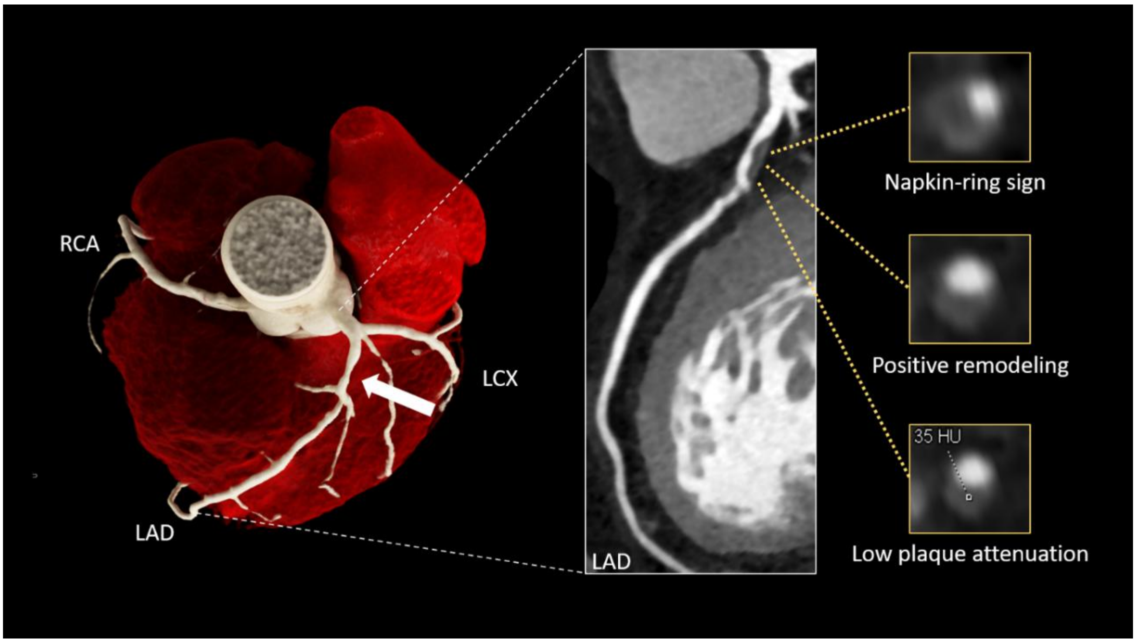

A 58-year-old male patient was referred for invasive angiography due to atypical angina. Patient was treated with hypertension and hyperlipidaemia. The figure depicts a proximal LAD lesion exhibiting 3 HRP features: low plaque attenuation <50HU, positive remodeling, and the napkin-ring sign (= hyperattenuating rim surrounding the hypodense lipid-rich core). HRP features were recorded for each lesion. The white arrow indicates the location of the proximal LAD stenosis in the 3D volume-rendered reconstruction technique The right panel highlights the HRP features, derived from a curved multiplanar reformation and the corresponding 3 orthogonal thin slice cross-sectional slices.

LAD= left anterior descending artery, RCA= right coronary artery, CX= circumflex artery

Supplementary Figure 3. CT based Management in the DISCHARGE Trial

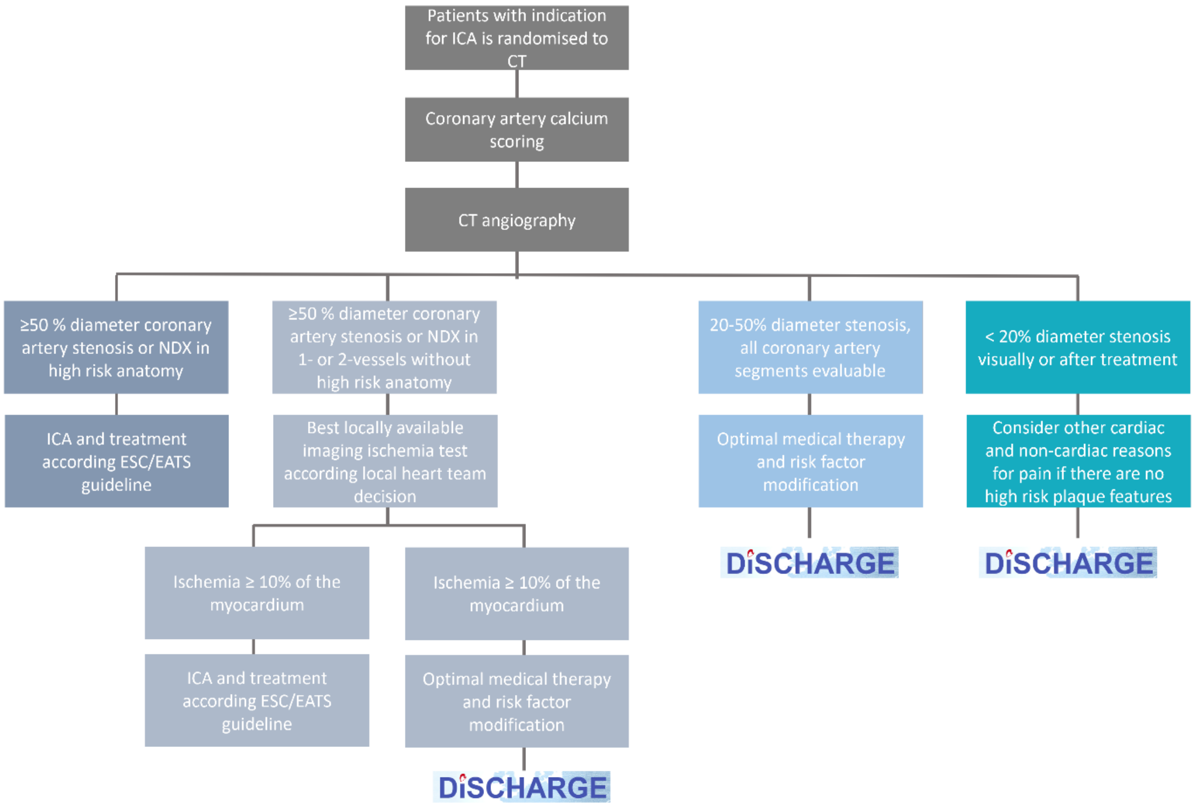

The CT based management protocol in the trial included the degree of stenosis, high-risk anatomy of CAD, results of ischemia testing to define treatment strategy based on guidelines available at the time of study conduct (Reference: Napp et.al. 2017. Computed tomography versus invasive coronary angiography: design and methods of the pragmatic randomized multicenter DISCHARGE trial (Eur Radiol 27, 2957–2968).
